# Supplementary figures and images for: RNA interference-mediated silencing of genes involved in the immune responses of the soybean pod borer Leguminivora glycinivorella (Lepidoptera: Olethreutidae)
Source: PeerJ. 2018 Jun 12;6:e4931. doi: 10.7717/peerj.4931 (PMC6003399; doi:10.7717/peerj.4931)

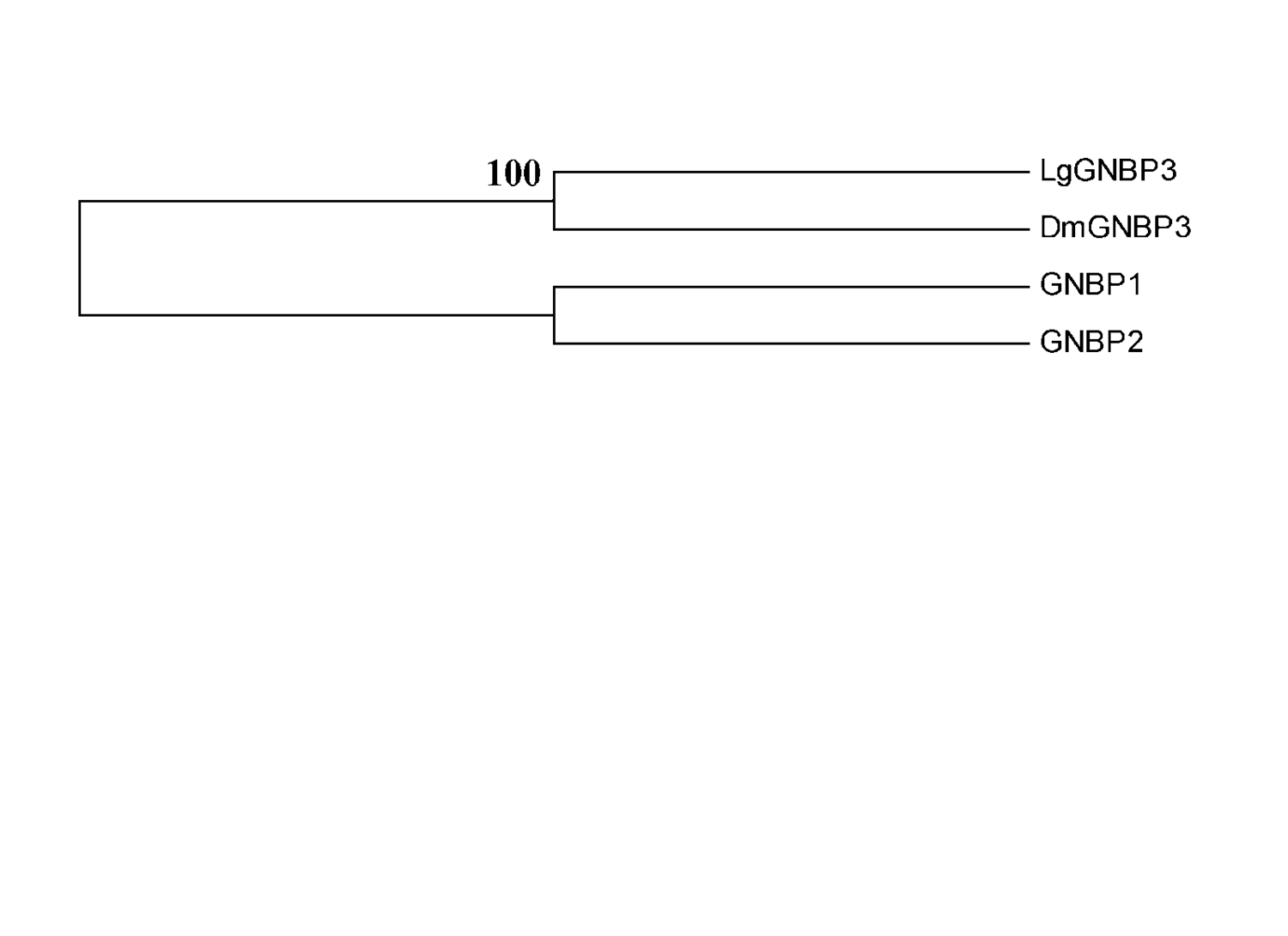

Supplement: Figure S1 — The phylogenetic tree was constructed using MEGA5.05 with a neighbour-joining approach. The bootstrap values (1,000 replicates) are provided next to the branches. The first two letters in each GNBP name indicates the species (Dm, D. melanogaster; Lg, L. glycinivorella). [file peerj-06-4931-s003.png]

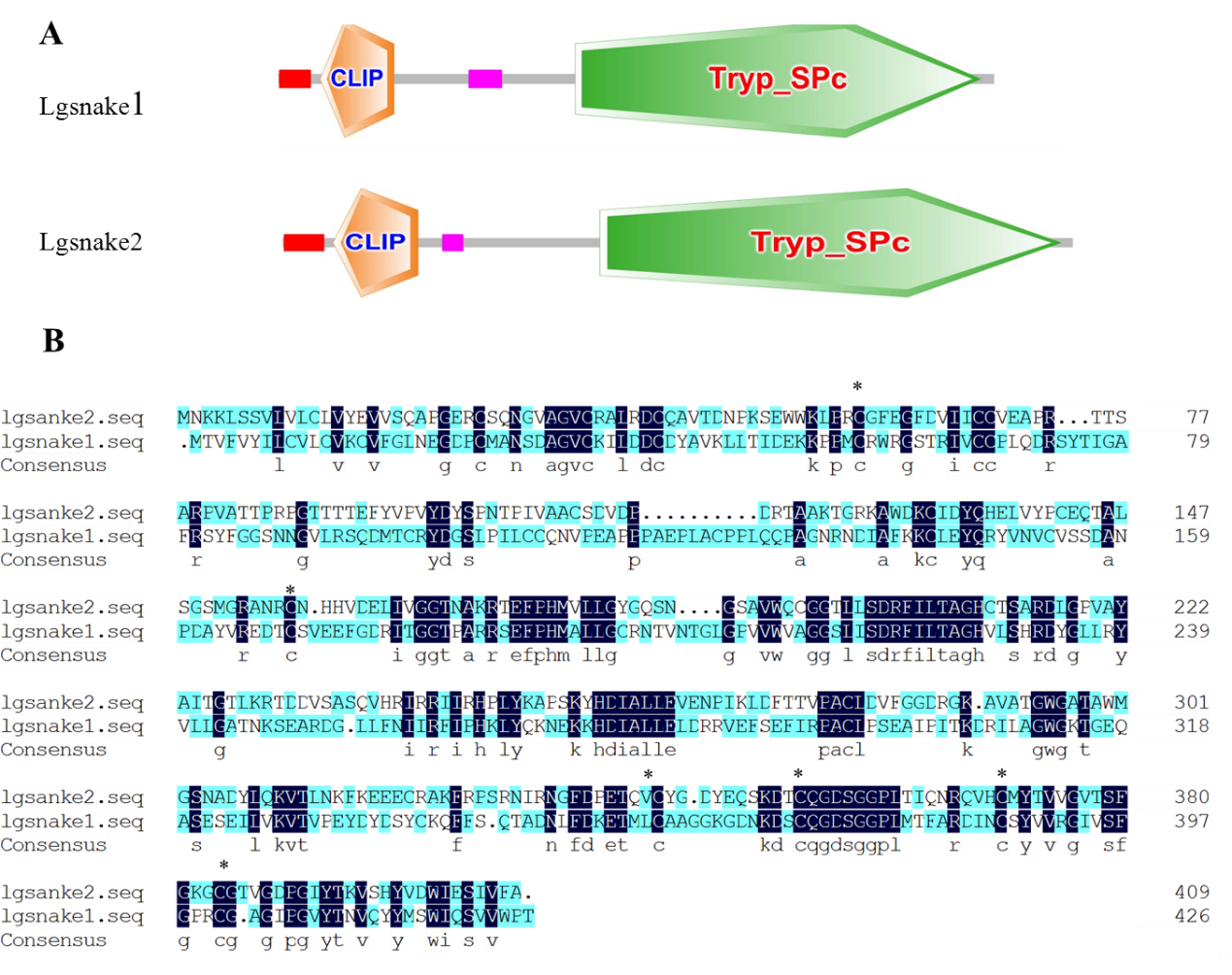

Supplement: Figure S2 [file peerj-06-4931-s004.png]

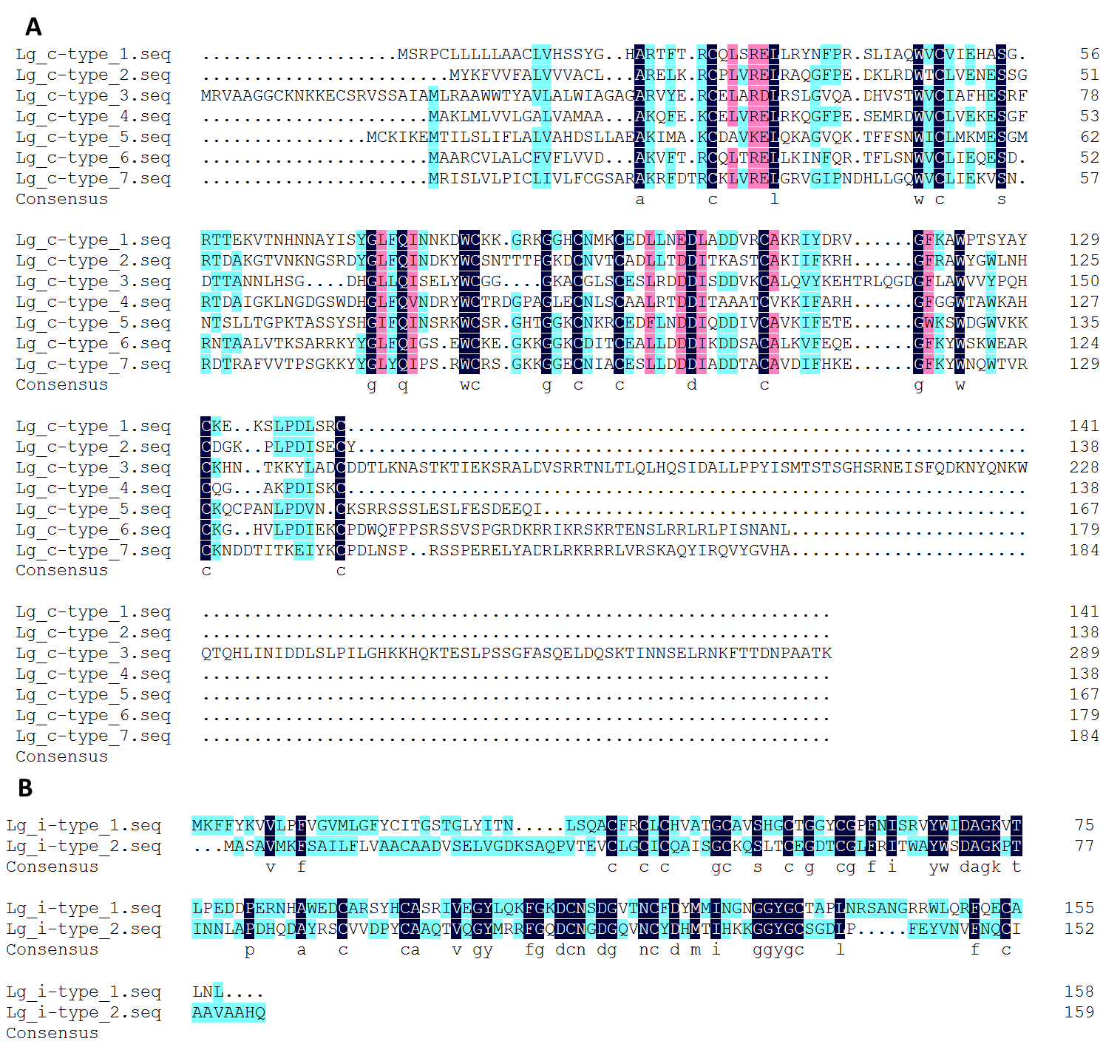

Supplement: Figure S3 — Alignment of multiple C-type (A) and I-type (B) Leguminivora glycinivorella lysozyme sequences. ClustalX was used to align sequences. [file peerj-06-4931-s005.png]
