# Supplementary material for: RNA interference-mediated silencing of genes involved in the immune responses of the soybean pod borer Leguminivora glycinivorella (Lepidoptera: Olethreutidae)
Source: PeerJ. 2018 Jun 12;6:e4931. doi: 10.7717/peerj.4931 (PMC6003399; doi:10.7717/peerj.4931)
Supplement: Data S2 — Protein sequence of the immune-related genes of L. glycinivorella obtained in the study. [file peerj-06-4931-s007.doc]

>LgPGRP-SC1 c58810.graph_c0

MIRVLSVFVIMILPKDFVLADCGVVTREQWDGLSPTRVEYLPRPVGLVIIMHTVSSPCNTDQTCAAQVRNLQTYFLDQLNYWDLGPSFLIGGNGKVYEGPGWLHVGAHTYGYNRKSIGISFIGNFNNDKPTPEALAAAKALIKCGMTKGHLKRDYKVVGARQLIATESPGRKLYQEIRNWPDWTDNLDGIKNN

>LgPGRP-SC2 c64599.graph_c0

MSLIAVLLVLIKLCAAQDNECGVVPMTQWVGQASQRTDPLATRATPVDLVIIQHSVYGACATDEACEKAVRGIREYHINKKGFTDIGESFLIGGNGRVYEGAGWSLVGAHTKLYNNRSIGISFLGDFRKTLPTSEALQAVKDLISCGVSHNFLAQDYRLVGHQQVVKTESPGTELQKEIQAWPHWAENV

>LgPGRP-SD1 c81439.graph_c0

MKAHTILYILSIIIFSANCLRIIYRGEWQHLRPIITSMEYEYTTASFALLYQTGGPSCFDEATCIARVKSIVESNMNEWGQIVFNYLIGGNGYVYEGSGFYEKIYFLRSLKYKCVTIAFIGDYTVDMPPKSARKSLEELLEFARDFQYLRRDFILLSSSQVEYNDEGPDSDTNLPSDKIDYHSNWNNSTLSI

>LgPGRP-SD2 c62396.graph_c0

MLDHNKLVFIYVITTVVICISQYAETSPTAQCCIFNHPQVLDFPIYLRDRWEAREPKKTPERLTIDPAPYIIIHHSDTPGCEDFDSCSKRIRSIQNHHMDVNGWDDIGYNILIGGDGGMYEGRELHIRGAHSPDYNNRSVGICFIGDFQSKLPNSKAINTAKQLITLFKNGIVLTSNYTLLGHRQTRATDCPGDALYNEIKTWSNWKDI

>LgPGRP-LB c73777.graph_c0

MSDAYESDCEELPVKTVKKKNRMRILVAVLLIGLIGVAVAIPTVMLTRKSSTSDPSENEVVTFDFPYVTRSEWGARPPVETLPLRTPVPYVVIHHSHTPKACYNRQDCIQAMKSMQNFHIDDRHWWDIAYHFGVGSDGVAYEGRGWPILGAHALHFNNISIGICVIGDWSNSTPPAEQIKTVKSLIAAGVDLGYIQPNYKLLGHRQVRDTECPGQTFFDAVKAWDHWSAFPASHEDLVNVPELSEEFREEYNKTLNAV

>LgPGRP-LB1 c68528.graph_c0

MAISGASYCLLLTVFCYVNAHPLTDDEPYPYYTREDWLATPATDVEPLSTPVPYVVIHHTYIPGACNTTEQCSASMRGMQEYHKSLGWGDIGYNFAVGSDGGAYEGRGWDTMGIHAGRANSHSLGIVLIGDWRVNLPPPKQLATTKALIAKGLKDGVISPQYRLIGHSQVMSTECPGGALLAHIATWDHYLPGHVEFKPATTNSTSNL

>LgPGRP-LB2a c65269.graph_c0

MREVQTLHQDDNGWSDIGYNFAIGSDGAVYEGRGWYRVGAHAIGVNNRSIGIVFFGDYVSDLPPPKSLQAAKDLIAIGVKAGFISPFHRLIGHRQVSATECPGQSLYSEITSWDRFLPDFDVNA

>LgPGRP-LB2b c62232.graph_c0

MAGKIIIFACLASSVLSLPVANQKNISIRNFRGFPRPFKYYSREDWGAKAATAVDHLSLPVPYVTIHHTYIPGACFNFQQCKSAMRGMQTYHQDTQGWGDIGYNFAIGSDGAVYEGRGWLAVGAHAYGANSRSIGIVFIGDYISDLPPPKSLQAAKDLIAIGVKAGFISPSYHLIGHRQVSATECPGQSLYTEITSWDRFLPD

>LgGNBP3 c76345.graph_c0

MCSNKNTMYLLYLCVCASVVVCYEVPPAKLEAIYPKGLRVSVPDDGFSLFAFHGNLNQEMNGLEGGQWSRDITKAVNGRWTFRDRDAQLKIGDKIYFWTYVIKNGLGYRQDNGEWTVTEFVYENGTKADPGNNPIYQPDKPLPTPVQPTQPPPTQPPPTCKPSPTVVLGKTVCKGEVIFSEEFDKSAVKDLKLWEGENKFPDEPDYPFNVYMTKGTMELENGQLIISPKLLDSMYHEGFVQESLDLSDICTGTIDTAQCKRVANGADILPPVTTGKITTKHKFNFKFGKVEVIAKMPAGSWLIPEINLEPRENFYGYRRYESGLIRVAFVKGNAEFAKKLYGGPVLSDTDPFRSQLMKQKIGIENWCSGFHNYTMIWKPDGMVMLVDGEEYGNIEPGEGFYTAARQAAVPHAGNWLRGSSVMAPLDQLFYISLGLRVGGVHDFADSADKPWKNKNNKAVLKFWEAKDTWYPTWHDASLKIESVKVYAL?

>LgToll-5a c79370.graph_c0

MNIRDAVRVVALLALATCVTTEMCPATTRVPVTCSLQSGTEIELSWSIREYITVECLDNGTFSCAELPKLSIPVLPTRVMIDNCVLPTYESLSCTFQALGASGATLLTLRGPHSFLKPEHVQGLHLSTLNLKGSTFDEPYTPLELPTSALAALSSLQKLVVTNARLNLSPDSLAGVSQLKHLELSSSDITGLNQSVFRELKSLLTLSLWGNDLRELQSGTFDGLQSLQSLELFDPLQSLDEGVFAAAENLRELRLENIELDELSQAALRDLNELEELIILEARRPLKVGLRAKDKSLPKLRVVRIERCGLETLPGDALAGARALQTLSLSHNAIETLPSEFLTDLTDLRALNLSHNALRSLEPALFQSLLHLEDLKLDHNRLEVLSGALFEPVPALRVLSARNNDLRALWSTEFRGLGELQKLDLAHNQLTLSESPETSISPLHAQTKLRQLGLGHNNITQVFDDWRNSLLHLELLDLRYNQIEVLEDPDTNFLSNVTVDFRFNRISMIKIEEENQYARVSPKDTRKGTLLIGENPFHCDCHTFRLLRLLANSSYEYIHPAIKLDAAVCATPPTLTGIKLREVPLDKLTCELPASKCPLNCNCTSRPARNTVELDCIEPPATAPMPDPPKDFNRIALRLRRAPLTLNLTLPVHDLQLTNLGLTEFPPGPIPTQLELLDLRNNLLTEISGLASGDYKLRLAGNPLACDCAHHDSINALLLSINRIEDWNNVTCAGGRYLKHIKDADGLCAEIRATWAASIGGALASLGLIAVVVTVTWLRYQNEIKVYLFARGWCGCLTKSGKEAPAKYDVFVSFSHEDEEVVVHKLVPELEQRGYRLCVHYRDWAPGEAITEHLSRSVREARRTLVVVSRGFLRSEWARAEFREAHARALREGRSRVIVVLLGELPAADSEDDELRAYLRTNTYVAWGDAWFWRKLQYALPHQPLGLQEDVPLVPLSSRHSDKLQI

>LgToll-5b c78627.graph_c0

MMDLKKSLVCVAVLLTLATSQSDGPCPSEATVGTQIELACFLPSGTEIEIDWRARKYVTLECDGKGSFACAELPKVSPKSVPPTRAMLKNCELPVNESLACTLQTLGAPIATSLTLRGIAAKHLTLDHVQGLVNLSTLTLVGSSVTHLPTAALSALPALRRLTVREASLHLTSDSLLGVSGLEYLELSASDIKQLNASFFRGQESLHTLSLWGNELRALQSGIFEGLDELDTLDLSSNPLQSLGDGVLEAAKNLRVLRLVDTELSQLDEPSLHNLNSLEELMILDGRKPLKIGSRSLALPKLRLIELERCKISVLPGDALSGARSLRTLSLSHNALESLPSELLRDSTELRALNLSHNALKSLTPALFQPLWHLEELNLDNNQLEEFSDDMFETLGVLRSLSARNNRLRTLSPTAFRGARSLQYLDLAHNELTLRSADTIETPIDGYMDFTDGRSSPMAELIAMQYLDLGHNQIQDVFYDWRNVMLNLKLLNLTNNRIEFLENPDINFLSIDAIVDFRYNQITTIQVSERELEARADDGESYAGSGRLLVDGNPFNCDCFTFSLLRFLNSPSYEHIFPVLTIDDAICASPPALANITVRAAPLAKLTCELPPPKCPDNCNCTLHPAEQAIKIDCMEPPLNVPAPYEFGFGLNRTVLRLQNAPPSLNISLPVHELVLSGLNLKAPPPGPAPPELKLLDLTNNLLTEVPPASGYKLRLARNPIACDCAHSDDLVALHKSTDRVWDYNITTCAHGGLLQLNSDANLLCARTKAAWAAGIGGTLALLGVLATVLAVLWLRYQNEIKVYLFARGWCGCLTRSGKEAPAKYDAFLSFSHKDEETVVHKLVPELERRGYRLCVHYRDWAPGESIAEHVSRSVLEARRTLVVASPGFLSSEWARAEFRAAHARALREGRARVIVILLGELPAPDPNAQEDDLRTYLRTNTYLKWGDPWFWQKLQYALPHRPLGDLKEVLPLAPGTISADALRAALAVHIEPKDKLSNGFQPRLLPPPAL

>LgToll-6a c77578.graph_c0

MLSMGLLAVAVWSWSWVGAGGASLTSRVAEAPQECEWQRVSGGPGEPTRVQLACSLRTAAGATDLLAGLSSSQAQRITALDLHCTDTLFFESSLDVGRRKEEGTELLSRFHNLKELRIESCKIRYVPSAVLSPLSGLRALSIRTHNTDWSAMSMEFHRDTFRGLTDLRSLDLGDNNIWVLPSEIFCPLYNLKELNVTQNRLQDISNLGFSDWGNGPTAPGKSCNTVLETLEMSHNEISALPDNGLSSLRALQKLFLQNNRISNVADRAFVGLSDLQILNLSTNALTGLPPEMFQSSRDIKQIYLNNNSLSVLAPGLLEGLDQLQILDLSVNELTSEWVNRDTFSGLVRLIVLNLSHNRITKIDALLFQDLNNLQFLSLEYNNIARIADGAFSNLKNLHSLSLAHNNIIEVDSSHFSNLYVLNQLFLDGNRITKVDLRSFENITKLHDLGLSGNQLSEVPEAIKTLRFLTSLDLGMNRITKVTTTQFEGLDDLYGLRLVGNKIEKISKDTFVALPSLQILNLASNNIDQIDDGAFASNQQLKAIGLDGNKLVDLKGIFTNTQPLVWLNVSNNELLWFDYSHIPSNLEWLDMHENKIEKLEDTYGVKESCNVKMLDVSHNKIRSIDEFSFPSSIETLVLNNNHIEKINPGTFLQKYNLNKVMIYSNKIKTLEVGAFAISAVPEEKDLPEFYISENPFVCDCTMEWLQRINQLSDLRQRPRVMDLESVRCSLTHSRAKMDVLLLEVKSSEFLCEYDSHCFTLCHCCDFDACDCKMTCPDRCSCYHDLTWNSNVVDCSAADYDHVPDRIPMDATEIYLDGNDLKELGNHVFIGKKRLQVLYLNNSNINTIQNRTFNGIESLRVLHLENNNLEVLRNTQFTRLQNLNELYLSDNKIKVIENDTFNYLPSLELLNLDNNGYVDYMPWRVITENNPRTRVSVDGNNWLCDCKDVAQLNQWLIKKSKDTETMMCYFAHGQPMNKTIATVAKECVSETATEETETETLKRLFIDSNDGVENYIPYIAVVLIIAIILLLMCALLFMFREDFKLWMHSRYGVRVFSSTPKDMNDNKNKRFDAFFVYNPRDEDFVTRAVSSELENSGHSLCLQHRDLQLIERRSGDSLVSASESSKRLIIVLSINFLQQEWYAGESRAAVQSAINSVNVRHRRQKIIFLVTTDLSAINIDPDLKVLLKTCTVIVWGERNCWEKLNFRLPDVDVTLPNRTLHNANNMKTGNREGFGRHGNLRYTAPPTSQDPWYKYGMAPPVLMMSSPMHSTSASAEVSARSTEDETCSVASSDGRPDGLPHHHSYVSIDNHQCEERPLRPLQQIPMSNTRPNNMRKTYFV

>LgToll-6b c78586.graph_c0

MLRNLCGMSSLKVWMCLVVVAAARARSAPPAPAGCQWDYSDAKLSESNFLTCNIKTIGSADFLFKNITTAQAFNINKLKLTCTDLLFFESSLHMNTGSFLGQLRKLEDLRVEYCKIRYVPATVLSPLRDLTSLTLRSYNTDWPAMTMEFHAESFRGLMELRSLDLGDNNIYMLPSEVFCPLFSLESLNLTNNRIQDISEIGFSDWGKGPIAPGKSCNTGLKMLDISHNNILRLPDNGLSSLRSLEVLNIHNNLINEIGDRAFVGLNSLKILNLSGNKLVAVPPELFQSSRVIKEISLANNSLAVLAPGLLEGLDQLEKLDLSRNRLTNDWVNRDTFAGLIRLVILNLSHNALARLDPKSFQDLNNLQVLNLDNNAIETISNGAFAELKNLHHLSISDNRIKSLNEHIFSNLFVLNQLYMDNNNIDTIHERCFENITYLQDLGLNGNNLNTIPEGIKKLRFLKSLDIGKNNITKISNTSFEGLEELYGLRLVDNQIESVPKDTFSTLPSLQVLNLASNKISTIEQDAFVSNPTLKAIRLDGNKLTDIRGVFSNLNTLGWLNISDNKLIYFDYSYMPAALEWLDIHSNNITKLDNEHNAQPNIRMLDVSYNALENVDERSIPDSIEILFLNNNKIHTINPGTFLQKRNLEKVVVTENKLRTVELSAFTLPHVAKHRSLPKFFIGNNPFVCNCHMIWLQKINLWNHMRQYPRFVDLDSVTCEVVNNKYGGKANLMDVPETHFLCSYETHCSSNCFCCDFEACDCKMTCPEGCSCFHDSNWSSNVIDCSNVGYTDIPEKIPMDATELYLDGNDFGSLNSHLFIGKKKLHKLYLNNSNIATIDNDTLNGLHALNVLHLENNHLTELSGGEFSQTKHLRELYLNNNLLTSVANKTFEGLSALRVAHLEGNKILDLDKKLASVTTLENVNVEGNTFTCTCDNVLLLQNWLKKHNQDPLEMLCVDENELVSNLTVFDVIDSCRDSSVSDNTIPTENQLYRYDEVSTINLNFVPLLAVVLISVILILLFGALAFSFRQNVRLWAHSKYGVRLFKSASIQESELDRDTMYDGYAVYSLLDDDFVSKVVAPEMEHSGYTMCFHYRDLQLAPENYLIEQITNAADSAKRILIFVSFNFLQNEWSKASFKAAVKHIITSIHPSIRRHRVVFILTTDVSALNLDLDFQNYLKSCNVLLWGEKKFWEKIRFVMPDISNLQWNKDTMNYNHNVCPNGRRHPSRYTASPTAPEHWYKYDAIPPTTPVGNVGINIEDDTSMLTNTTLTSQIPDGENIHHSYISIDTQTYEQPYGGRPRPPHTLRKHPVHNSPSMLDEQGYLQPRSSVHECLPQTHIATHR

>LgToll-6c c76351.graph_c0

MALQKSSSRKQAFSHILFLFVVGLNVADQSSLPLKYEAPDDCQWWLRGPNDSHEVSLTCKLRTINSEFDTTNFSVIPSEHTTSLRIECNEEMMYKSSLDDRSFAHLIKLRELVLDNCKIGRWPPGVLSGLRDLRNLTIRTKNTEWAAMSLEIASESFAAVRQLEKLDLSFNNIWSFPENLFCPLTNLVYLNVSSNRLLDVSDLGFREHAVHQALISEQDGPVPSSSLPHASCSLDIEVLDASSNRFVLMPENGFMSLRRLKELHIHDNEISMVSEKALSGLKQLQIIDLSNNKIVALPQDLFKDCKTVIKEIYLQNNSISVLSPNLFANLDQLLALDLSNNHLTSTWVTENTFRGLIRMVLLNLSNNRLTKLDPKIFKDLYTLQILNVQHNSLENIAADTFAPMNNLHTLILSYNKITHIDAYALNGLYVLSLLSIDNNHLEDIHPEAFRNTSSLQDLNLNGNRLKKVPIALRNMRLLRTLDLGENQITLLEEPGFVGLHNVYGLRLIGNKIENISKDVFTDLPSLQILNLARNKLKKIDINAFDTLNNLQAIRLDANQLTDIQGLFANIPSLLWLNVSDNQVEWFDYAFIPTGLQWLDLHSNNIKELGNNYRLNKELRLQTLDASFNKMTKISTFSIPDSVELLFLNDNQITQVEAQTFVGKTNLTRVDLYANQITSMDLNALRLTPVDPGRPLPEFYIGGNPFQCDCTMEWLQRINKLDHLRQHPRVMDLESIYCKLLYNRERTYIPLIEAESSQFLCTYKTHCFTLCQCCDFDACDCEMTCPSNCTCYHDQPWSANIVDCSAAGYAEIPNSIPMDATELYLDGNNFGGLTSHAFIGRKNLEILYANNSNIDALYNNTFSGLKRLKVLHLEKNNIKELLGFELSPLENLRELHLQDNKIHYIDNRTFIELRRLEVLRLEGNNIYGFAVWQFTVNPYLVEISLSRNPWSCDCQYMNKFRNWFKNNFGKVEDAKKITCVFDNVTNAVGPLMADFNSTICTSHVGGSSSIIENQVINDYLPLLLISLCIFIISSALICGVFYWRRELRVWIYYHCGFRMCYKSTAFDDEADKDRLFDAYISYSVKDEAFVAQMLAPGLESTDPSFRLCLHYRDFNASAYVADTIIEAVESSKRTIIVLSKNFINNEWCRFEFKTALHEVLKERRRRLIIILLGELPNRDIDPELRLCLKANTCIEWGDRQFWQKLRFAMPDLRKCQYHRSTVNIYASVSPVGAGRAPAPPPPPPPGKLPPLLGDGLALSASVHARDAHAHRMPPHAQLWA

>LgToll-7a c78030.graph_c0

MAIPNSVELLFINNNYLNNIHVNTFYEKKNLTRVDMYANEIVHLDQNSLRLAPVPTNKTLPEFYIGGNPIKCDCTMEWLQIVNYTTATRQYPRLMDLENVLCKMTNTRSGTHVPLTNLKSSDFLCAYETHCFAICHCCDYDACDCEMTCPQNCTCYHDPLWNTNVVDCSGQSSIEVPHKIPMDATEVFLDGNNIKELQNHVFIGRQKMRSLYVNNSNVDNIQNRTFAGLNALQILHLGNNKLKELKGYEFHQLSNLKELFLQNNLISHIVNVTFLTLKSLETLRLDGNRLVDFSVWTFNNNPNLKALSLGNNLWSCKCRYLQELTAYLAENAQKIIDITDVWCWNGDAKPPQKKELNLNGTACSDYYADNSVIGNMLVSNYVPMMVSTLTGFMLILLALVILFLFRDSLRVWLYTNCGIRVFSFAGAFEESEKLYDAYVCYSPKDEEFVVQSLAAELENGNPSYHLCLHYRDIPHHGAQYMQCAPPVVEAAEASKRIIIVLTRNFMQTEWSRYEFRQGLHDALKGCIYKLVLIEECTVVADAICDPDLRPYLKTGSRLRWGQKRFWERLRYMMPDSSQPNHKRRSHNYRKNINTYTLDSSVPNGGNRTMPYPDKSPVIGGGQGASAPPEYSSEVRQSPSVVRQTQGVVYGPDGRPISDHIYSSIDSDYSSLEHGMAPGRRRDIRQWPPPPPLVDTGNTVQAYLV

>LgToll-7b c78155.graph_c0

MSIPNSVELLFINNNFIASVETDTFLQKRNLSRVDMYTNEIESLELNSLRLARVADDRALPEFYISGNPFRCDCTMKWLLLINSLNSREYPRVMDLENVICKESYVRGVKYLPVSSLHIKDFLCKFETHCPEGCNCCDSINCDCKTVCPKNCSCYHDSTKSTSVIDCSLKQLKLVPLNFPSDSTHVYLDGNVYNELNGTVFDPMPKAVVLYLNSSNIAHIGNDTFNRLSELRILHLDNNRLTRLRGLEFAKLSNLKELYLQNNIIEHISNETFYSLAALEVLRLDGNRLVNYGLWHLDNNKKLQTLFVGGNRWSCDCKYLQGFLTYISQNVEKVVDIGSVWCVNGPEPARKPLNLNVTVCSEISDSSVISAFFVSNNLPLVASVLTGFMLILLILVLAFTFRYACRMWLFSNCGIKLSPLAGAFEDADKLYDAYVCYSPKDEEFVIESLARELENGYPSYHLCLHYRDVPQFEATYAQFPDLVVEATEASRRIIVVLTKNFISTEWSQLEFRQALQRALRKNPHKLIIVAAGLVPRDPELKSYFKTGLEITWKEKRFWERLRYAMPMSKRRSHKLKRLNYGRSSNTYTMDASVLNSTCQTLCGKSATSAERSPADRPLSEHIYSTIDSDYSSQDYHSRHHQPHSVVMQHTVQTYLV

>LgToll-8 c74702.graph_c0

MCCTWFTSALLVLSAVLTTWSASLSATTGARYQAPDECRWTTDDDGSGVALQCRLRTINSELENTNFSAIQPHLTVRLRLECSDALFFQSSLSPGSFRQLVELRELTIEYCKIGNLSDGAFTGLRELRNLTIRTHNTDWSSMSLEITPTAFSRDVQNLERLDLSENNMLVFPEGALCTLRNLEYLNMTGNRMRDISHFQFSSAHRHPTEKCGDNILVLDLSRNVIDTLPPNLLSGLKRLQKLYLQGNALNSVADRALEGLISLTTIRFSDNQLTSLPPELFSDTKELKEIYLNNNTITVLAPGLFSDLLQLLVLDLSHNELTSDWINTSTFTGLKRLVFLDFSHNRVSKMEVALFRDLHNLQILKLQDNFIEHIPENVFSPLNSLHTLILSNNRLTAIESYAFSGLHGLSVLSIDSNRISKIHPHSLRNCSALQDLHINGNRLDEVPIALKEIPQLKTLDLGENLIVSIENASFMTMQQMYGLRLTENNIGNISKGVFDKMTSLKILNLSRNKIHKIEAGAFDGNINLQAIRLDGNYLTDIGGLFAKLPNLVWLNISDNRLEWFDYAMIPTGLQWLDIHANRIAELGNYFEIESQLSLSTFDASSNRLTEITGSAIPNSVEMLYLNDNLISKVQSYTFFKKPNLTRVDLYGNKITNLDPNSLRISAVPQDKSVPEFFIGGNPLECDCTMEWLQKINTGNRARTQPKLMDLDSIYCKLLYNRGNAYVALVEAASHQFLCKYDFHCFALCHCCDFDACDCEMTCPNNCTCYHDQSWSANVVECSNAGYVNALPERIPMEATQLYLDGNDIKMLPSHAFIGRKRLKILFLNSSNIESIQNRTFNGLKELEILHLDHNNLKAIEGQELDGLDNLRELYINNNQIRHIGKEMFNHMSRLKILHLHNNRLTMLSVWQINSIVTEITLSYNPWSCDCEYTEMFREWMKRVNSVTDISNVKCIYSQGNNTEMVVYRESAFSEPESGFVIASENGTICTGLPSINNSINGNLTATKTIISNEDVQDYIPLLVATLGAFLLVSFVSMIVFIFRQEMRVWFHSRFGVRLFYRASDIDRDDREKMFDAFVSYSSKDEAWVAEELAPMLERGDPSYKLCLHYRDFPVGGYVADNIIQAVESSRRTIMVLSENFIKSEWCRFEFKSAHHQVLRDRRRRLIVVLLGEVPQKDLDPDIRLYLKTNTYLHWGDRLFWEKLKFALPDVPNNQRCRGGPSPGAVGGVAMHRHHHARSHLGALPPPPVHGAHPVLPPHPAHNPHQAPPRASPRTISVHV

>LgToll-9a c73304.graph_c0

MNIYAFVICNCLAMVWGQQVQPCLTGSMTDIQPWVDRNGRLKPEAKPMSIDVSESRQPMATLELQLNQDTRNPRKIRILSMARCSLRTVPEVFSLQDANQRPLSEVIEYLTLYGNKFDDHQSTGDRYDSFLNATEARVLVQSIAYDTREASWPGSLKNTRFKNLRELDLRACAIATLSIGTFQGMEKLEALYLSENSIVYIDVQAFSGLKNLAHLDMSRNYHYDDFGNYKSLVLDSMDAIGDLNKLVSLDLSYTRLSLGNLMIFNSFGKTFQRLSLCETGLTKLRDNMFRNTSIRFLDVSGNNGILNQFGALSGLENTLEVLYAVKVGLVNLNVLDGFKRLEILSVQNNEISLFTNDVTKTLVNLQILNLDYNRMTTWLDPKFSHMKNLKYLSLRKNNINMINENMLQDLKNIEYLGLSENFVICNCHTRELFETALITDRAETNRSVLISQYKKGGNNKFSSYHTGFEFYNAIIDKRTNVNKACEKDKTCDTEIHNDNITASYVLVDYDPNLYVCMQMIEGVYKPFSQPMSCDISPRDVDFEVVMDKSRMKLLALIIIPAILFPMLYYMFIFRKNFRYFCITVRNSALLSMINKDKIIDDSRIFNYDVFVSYCNDDRGWILDQLLPHLETNCGVSVCLHERDFQVGLSILENIVSCMDRSRSIMLVISKEFLLSQWCQFEMHLAQHRLLETRREDLMLVLLEEIPRRLRPTTLHYLMLTKTYIVYPNGAGESVKKDFWRRLGRSVTARRPDHENDSLA

>LgToll-9b c76475.graph_c0

MFGARYLWLLLCYAQCARANLDTDFEGVTTILSQNEDRSVDLPVELGTDAASGCICRASKHRHVIVCFGNYECKTFPKIEYKSKSLRVRTTAIRSLRKGDLDTLHHLKILEVEANHMLAHIESGVFLNMSNLQHLSISYNIGLQTLAQDTFEGLTNLHNLTLVNNGFTNILDLTPAFKVSILPSLRGLDLSENCFEHIPEEAFIPMVGTSLKHLEIHLCSLDFIHPNSFLPLRSLKELHVGENDLSSSLIGNFLLKMMEEGINLTSLDISGMGFRKQPPQRLINIIAQTTIKSLNLAQNQFEVINDAAFPRMENIEVLDLRRVSAIFIGPNTFDDTRFPNLKVLLLGGNNLPGDHETHLSNQLILLDLSSNKGNAANPLYYEIGKDTFSQSKKLRVLNLSYNRIKTIFEYTFRGLEKLRILNLENGTIYFIGTDTFKPLKQLEMLNLANNPLVANENLTSAQFNGLNELKILILMNCGIKRFYDNDNIFEMMPNLTHLNLKNNQLYYITAETIKPLKFLQVLDLSENLIISWWKPLFLAAGVAPKRLYLMNNKISHFSVSMMQDISYLLEDGRNSTAEVDLMDNIFVCDCSTMYDTYIWLEQNGSTVLKDYFAQSQFQCSSPDVWEDRKVSEYLSSVKKLHCLVYQKISNIMVLVWTAPSLVTIILIVSSVTIIYRYRIYIRYWIFLAKLALGRTLIAKTQKTENMVEKTYKYDSFVSYCADDRDFVMEMVSELESPPSCLKLCVYERDFEIGTFISESIVTSINESRFIVLVISNSFVRSQWCRWETQLAEYHRLFLEDGSVYDPLVLIKIGEIDNKYLTTTLKYLLKTKIYHSWDEKNPEEFWKKLKNVLSKK

>LgChaoptin c77948.graph_c0

MRALSLLPMLTELSFSNNKITDASMVGRATRDLPSLSNLNLNNNLIDKLNEGAFVDQGMLESLFLSNNNINVIHHGAFHRVPKLRVVDLNYNNINRIHPESFLQQSGSGVEELNLIGNQITHISEFRALLDALPRLRFLDMSENLLQDIPRGALRGHPSLERLHLNKNNIKFIQADAFMAMPALRELHLSNNSLTDFNEGPFWNLPALKGLDLSFNYFQRLQPKLLYNLPSLRRINLSNNQLTMIDPITFIESPLLEYVNISGNALVSIHPATFRNLASLYEIDASHNRLMEFVPGLPRGLEQLCLQRNQITSLPVPPSPDLDLPSLRTLDISINGIQKMPRGSMKTLHNLRRLYMRRNGLRQIDPTTFGDLERLEMLDLGENQIISVHPKSFTKLQYLKQVNLHGNTIENFDFMAVQDNAALSTLDFSKNRLKSISPNIVNRALDIEILNISSNNLVELPISLNMLPKLRLLDASYNHIKFFDGNAINNIHTLKEINMVNNKVTELRSGSFEDLRELETIHFESNQIEIIHPKAIINLPNVISIHLSRNHIIDLPDRVFSNLPKLRIIELQGNRLQYISLRAFENLPLVQYLNMSNNQLTNIDNSGLRQLASLEVLDLSFNKLNKITRASFQYMEWLVELNLDNNLICYIGGQPFDFMPRLKVLSMQHNKLTTVGEPVFAKLRNNIAILDIDGNPLICNCRIVWLKSWLAESSSIGPKCADGTYVKGMPFSSRDCVNTPTGDEDLRSCVALENEALLPNLATSQVFSTMDKIKDYTQQIKNNYHINKINRPSPEESEYFYDEYVDYPYNETLIDGLNEISAQNKNKSHYTPGDTPTLYAEMKNKTNNNPPPNAGSSGFTFFGMPLPTINMGQLLNTGRKVDWQENKQQNPTDKYLHEPPKFETGGFSPILPTTATGFKPMTNPNMNMTQNAIANMGDAKYSAKGSVQHSVVETSTKPPVNTVQRNSTHQKMKSEIHEIQAFLNDDNSTHVAYNRTKAIEEEKIDPNNISKYNLMESNLTVNHITEKEGLLITTDTSNDMSLQAWLETSTQPFSGSSVTSKPPIKKHIENNPPTALSAILVPNNEELARRNYTKRPATITKVTMPHAEHYDNHDNYLPVINREGKTRFSELPTIGNTKTRQAEDRDWYYKNYNNSNLEPYVAPGVHNSGGNIVKVKIYIVIVGLCFKLYT

>LgCSIT c73390.graph_c0

MATILRHILRTKPPNIMFRRWEGTEKKVVVYDPFVNKPQKNKESYLEVIKMFEGRDTRRRGHVEFIYAALGRMKEFGVQKDLEVYKALVEVLPKGKFIAQNIFQAEFMHYPKQQQCAVDLLEQMEDNGVMPDSELEQILLNTFGRRGIPLRKYWRMLYWMPKFKNLSPWYLPDELPADVMELAKLAIQRITSVDPDTGVEVWQTEEIEASLDKTWIVSAQSDAQKILLAEQAEDEPLVVRGPYKVCIRDQFVTYFLLLGKIRPEVKDESDPDDVSNLKKPSGIPGFIGHTTLPSTRSIVHEQDDGTILSVCATGTSSRDSLLSWIRLLERNGNPLLSNIPVIFTLTAPPNDITIQETQPATGNEEKVASTS

>Lgotollip1 c74346.graph_c0

MTSTIPNDGDRNEERRRRVLLGPLPAGFLRADGDTAADNAMDADYQAALALQQQLCGAQVPAAGPPLTARLSITIAQAKLVKNYGLTRMDPYVRLRVGHCIYETHTDPSGGKTPRWNKVIHCLLPPGVNSVYLEIFDECSFTMDELIAWTHITIPQAVLNGETHEDWYPLNGKQGDGVEGMINLVLSYSVGPAAVATFPPVLVVPSTGLGYAAMPVYPQQPPLQPPRAHPPPQPVVTQEQIQQIEEMFPSMDKEVIKSVLEANRGDKDATINSLLQMSE

>LgFadd c76721.graph_c0

MVRVEVRPIHATQCRTMATNYSKLKDLITLKVSMSDMHSQILHAMKGLYQNDINSARRYEQITTIGQLLKVLEVRDVLSEENILPLQVLAQRLPDNMEIMNSISCYEQSRGPKQNINQYAPSLSDPVWRSMSNIQRMAYCAWCVAHGLWRITCGICRVAYRIWRVMYWTWRVCYVLWRITYFLWNILTRVLNASHHHQNNMRIMHSQMVYVRRNVKG

>LgDredd c73745.graph_c0

MCLPKKENPDLVCFILFSSLTMMCIDASVQVEENAISDKNLNAINLEVITMIEKELEVYDLISLVFLLYDVPDTALDRLIILQRISRDIEGSHMNLLFDWALSAQGRPTWRQQFLEALTVCQLYNVIRKLGFNVSTIKKLYSSNEDSPHINPMKKVLYQLCENMTSNTFENFKKTLVSFDMNVLQYDTCELVLLELMSQKFITLRLLNFERSVPRCECQIEQLAKIIDREPELKRYATILRSTESKLNVKLESPSTSVEKPNLNITDNNTETCDYNEVFNMIEKLSLEDIPIDLKSDRQPSINLNDRYKITNEKRVGICCIINQEVFYPSKESMEYANENALPKRDGSTKDKVMLEKTMRALNFDIITRDNLNRNEMIAFIQEVLKKVSAEDSVFMLCILSHGIRGCVYAADSIPIKVDYIQSLVDEKQSLRGKPKVLILQACQEDDTTLPLVPDSPHNMRKTDFLVCWATAPELVAYRLSSKGSFFIQILCEIISIRGKREHLLDIFTIVNEEVVKKCTLWGVEQAPIVNHTLRKKLYLQMCG

>LgIap2 c75936.graph_c0

MNLEINRLNTFSNWPMGAPVDPIRIAKGGFFYTGQGTEVECFSCGGKISQWNYGDQVMMRHRRMDPNCPFVVNPQMAGNEPLVLDRAFTAQSANVPVPAPVPEELVTTSQDAGPTEEDEMYKSDALRLLSFINWQDDSISREALVSAGFYHAGEGRLRCAWCGGELAPFRNLGSLGAPVDIHRRYFPRCDYALRLDNAYRASNISPPFSPPHTPQVESPRNSVMSEGALHNSRLLARSGQNCTQLGVVGVSGPGVQHPTLASLAARLATFAEWPQGRPQGPETLAEAGFFYTGQDDQVCCFYCDGGLGKWESSDEPWAEHARWFPGCGFVQLVKGAEFVAQHRGTQPPRQTSDDRSVNTRQRNPSVNFPVNESQVEEQMDSQQALVALGAGLDAARVRRAIRRRLQTTGLGFTSSEALIDAVLDEQLNEESWGVNTNQRFARDILAEALREFAPAAGLIPSPDCDQTSQSESSSRSRTPNEPQPSQEVTVAHADTSQTLRDPVRLKPTVSNDRVSSDRVDRVESEKENGPVRKKLSLEEENRQLKEARLCKVCMDSEVSVVFLPCGHLVSCARCGAALPSCPLCRSPVRALVRAYLA

>Lgsnake1 c73257.graph_c0

MTVFVYILCVLCVKCVFGLNEGDPCMANSDAGVCKILDDCDYAVKLLTIDEKKPPMCRWRGSTRIVCCPLQDRSYTIGAFRSYFGGSNNGVLRSQDMTCRYDGSLPILCCQNVPEAPPPAEPLACPPLQQPAGNRNDIAFKKCLEYQRYVNVCVSSDANPDAYVREDTCSVEEFGDRITGGTPARRSEFPHMALLGCRNTVNTGLGPVVWVAGGSLISDRFILTAGHVLSHRDYGLLRYVLLGATNKSEARDGLLFNIIRFIPHKLYQKNEKKHDIALLELDRRVEFSEFIRPACLPSEAIPITKDRILAGWGKTGEQASESEILVKVTVPEYDYDSYCKQFFSQTADNLFDKETMLCAAGGKGDNKDSCQGDSGGPLMTFARDINCSYVVRGIVSFGPRCGAGIPGVYTNVQYYMSWIQSVVWPTK

>Lgsnake2 c77617.graph_c1

MNKKLSSVLVLCLVYEVVSQAPGERCSQNGVAGVCRALRDCQAVTDNPKSEWWKLPRCGFEGFDVIICCVEAPRTTSARPVATTPRPGTTTTEFYVPVYDYSPNTPIVAACSDVDPDRTAAKTGRKAWDKCIDYQHELVYPCEQTALSGSMGRANRCNHHVDELIVGGTNAKRTEFPHMVLLGYGQSNGSAVWQCGGTLLSDRFILTAGHCTSARDLGPVAYAITGTLKRTDDVSASQVHRIRRIIRHPLYKAPSKYHDIALLEVENPIKLDFTTVPACLDVFGGDRGKAVATGWGATAWMGSNADYLQKVTLNKFKEEECRAKFRPSRNIRNGFDPETQVCYGDYEQSKDTCQGDSGGPLTIQNRQVHCMYTVVGVTSFGKGCGTVGDPGIYTKVSHYVDWIESIVFA

>Lgserpin1 c74306.graph_c0

MRFVLFVGLLLMAISQLANCTPAEYSSYSVTDDAVSDDGSILVLAFNFERIKAKFSEFTDLLGKMIALLMAEPDYCEATKEPQTTDSSLTTIEPDFSTEFDVKSVNSFASKFIKSLDLSHQNYVCSPLSVWILLSIFYDGARSTTAEKLGDVLGIWEKVSTYQLSDNFRGFRQTEIVLANQIYVANGVRLESEFVQTAHILNDVSIDNLDFSNSNRAANKINQWSKNVTHGRITDIVKPGDVGPDTDLVALNAVYFKGTWKTHFNSTKVQNFYLPGGKIKEVPIMRVESKFLFGYLEEHSAYYVEIPYKSENDHPVSMFVILPYTPDGMEYLKTQEFDIQELRRRGSVETVEIFLPKFNISTNLDLENSFENLGLGELFENPDFSGISKSSSLAFSKATQKAFIQVDEEGTEAAAVTDFIVSRSMGEDLKFEANQPFIAKIAAVEENLTFFDIYYEAEEYSVADLTYEVV

>Lgserpin2 c71704.graph_c1

MEKRKESLSDIKLCRFCLTQDSSLTSLYDRSRDPILVTLPLKIMACVSIEVFPSDKMPSYICERCRVFMDICYDFKQICRRADESCLQFVQNGVPISAVNWPASLTKIFQLTKKTAPRPINTVVEGGATIAVTSQLSQDMSENDNEEEETFNIKIDASHDGSKRIKVTPSGDKESSSAKKAGFSTVSKLSRHVRAHAGDRAFPCKYCSKSFVKSHHYTRHLRLKHEEQTRQSRGVDAEFRCEQCTESFGTQDELYYHSAIHATQNLTCPLCQEKFENVDAVTTHIRTHVNGIEFMCDYCELVFTSKEKLDAHMTAAHDDETWPGKEMDESSLEADAMDADVDDDDDDGLNVKEEGDHMVVEIKKADGYMLKKDVEVGQLVTTNSEGSGVSVYTDSDAVDSAEPPREAPREPARRPPPATKSEATSSRLPTTSAANERISILRKAEAMKRKAVAKEDSDVPQKKERAAKVDSTTSSAGSSDKSLRMLEKELQELKRTTTRVEGKPVKTAEAKSKRQPVHTSTPKLRSGDEKKAQSTPKFNTSVEKKSAERRVLNKEPKEAVARNTVTSSKDDKNVSSTKEDKPADREDSKKNREDENQPEKPKITDKAPEKEDKGSKEVKGKVTKEDVPSSSKNEASSSTKAEKSAPAKNGEKTVKNGDTSGSEDTSIRRSTRPSKIKNYASMIRDRTRMIRDEDDDEEVSDMEEEYVGPEPDSQEQPSRKSLGKRQKAATPAPAASTSVTPAARRRGRPRKEATPKESVEDAEVETPTVKEAEKKPEEPTKEPEPEPEQTNKEIPTEVKATTPATSTESQVNTLMSPTGQTLKKVPVKALPPGVKPLPLPLNARRDLCQMQIGKKMVKVQKIVMTKAEVEAMAKKGLLELKDGTMVLKQGIKLPGGDPAVLRSGLVSQGGE

>Lgserpin3 c67714.graph_c0

MRFLFLIGLITWHVTDSNAMAVVGEKSEGLKAVADSANTFAPNFFKTVAESKKGNLICSPISASMVLSLATYGARGNTEKQMRSVLALPEKDEVAKTGFQAFIDNLNNYNAVELRMANKMFVADGIEPKTEFKAMAQTGFRSEAQNVNFAKSAEASKTINDWCEEQTNHKIKDLIKPDAVDASTAMVLVNAVYFKGNWSSKFDPAKTQDRPFHIDETQTKNVSTMFRSGSYRYGKLPEVKASFVEIPYKSSGENDAISMIVILPDEINGLAELEKNIEKVKLETLLRGATRDVNLYLPKFKIESDLDLNPILSKMGMSDMFTNAANFTGIVDAPPLKVSSVVQKAFIEVNEEGSEAAAATGVVAVTFSLVIPEEPPVDFIVDRPAHFIIATNSYDYPNSILFSGSIHEPMTN

>Lgdefensin1 c81281.graph_c0

MKVLVVVFLLTVFVTVMSAPSPQELEGGKVEIDPENKVYIRHRRITCDLLEGSGNKYCAAHCIALGKTGGTCNDGVCICRRETISSLLPDQAKKVKTNIVDLWNF

>Lgdefensin2 c34040.graph_c0

MDFGYLEVEELLLTPTVDVREAPTMAAPVAAALALLVAVGRVVHGSPNAAANVHVCLNRV

>Lg i-type 1 c67664.graph_c0

MKFFYKVVLPFVGVMLGFYCITGSTGLYITNLSQACFRCLCHVATGCAVSHGCTGGYCGPFNISRVYWIDAGKVTLPEDDPERNHAWEDCARSYHCASRIVEGYLQKFGKDCNSDGVTNCFDYMMINGNGGYGCTAPLNRSANGRRWLQRFQECALNL

>Lg i-type 2 c75841.graph_c0

MASAVMKFSAILFLVAACAADVSELVGDKSAQPVTEVCLGCICQAISGCKQSLTCEGDTCGLFRITWAYWSDAGKPTINNLAPDHQDAYRSCVVDPYCAAQTVQGYMRRFGQDCNGDGQVNCYDHMTIHKKGGYGCSGDLPFEYVNVFNQCIAAVAAHQG

>Lg c-type 1 c71261.graph_c0

MSRPCLLLLLAACLVHSSYGHARTFTRCQLSRELLRYNFPRSLIAQWVCVIEHASGRTTEKVTNHNNAYISYGLFQINNKDWCKKGRKGGHCNMKCEDLLNEDLADDVRCAKRIYDRVGFKAWPTSYAYCKEKSLPDLSRC

>Lg c-type 2 c57709.graph_c0

MYKFVVFALVVVACLARELKRCPLVRELRAQGFPEDKLRDWTCLVENESSGRTDAKGTVNKNGSRDYGLFQINDKYWCSNTTTPGKDCNVTCADLLTDDITKASTCAKIIFKRHGFRAWYGWLNHCDGKPLPDISECY

>Lg c-type 3 c56706.graph_c0

MRVAAGGCKNKKECSRVSSAIAMLRAAWWTYAVLALWIAGAGARVYERCELARDLRSLGVQADHVSTWVCIAFHESRFDTTANNLHSGDHGLLQISELYWCGGGKACGLSCESLRDDDISDDVKCALQVYKEHTRLQGDGFLAWVVYPQHCKHNTKKYLADCDDTLKNASTKTIEKSRALDVSRRTNLTLQLHQSIDALLPPYISMTSTSGHSRNEISFQDKNYQNKWQTQHLINIDDLSLPILGHKKHQKTESLPSSGFASQELDQSKTINNSELRNKFTTDNPAATKK

>Lg c-type 4 c50330.graph_c0

MAKLMLVVLGALVAMAAAKQFEKCELVRELRKQGFPESEMRDWVCLVEKESGFRTDAIGKLNGDGSWDHGLFQVNDRYWCTRDGPAGLECNLSCAALRTDDITAAATCVKKIFARHGFGGWTAWKAHCQGAKPDISKC

>Lg c-type 5 c39033.graph_c0

MCKIKEMTILSLIFLALVAHDSLLAEAKIMAKCDAVKELQKAGVQKTFFSNWICLMKMESGMNTSLLTGPKTASSYSHGIFQINSRKWCSRGHTGGKCNKRCEDFLNDDIQDDIVCAVKIFETEGWKSWDGWVKKCKQCPANLPDVNCKSRRSSSLESLFESDEEQI

>Lg c-type 6 c62097.graph_c0

MAARCVLALCFVFLVVDAKVFTRCQLTRELLKINFQRTFLSNWVCLIEQESDRNTAALVTKSARRKYYGLFQIGSEWCKEGKKGGKCDITCEALLDDDIKDDSACALKVFEQEGFKYWSKWEARCKGHVLPDIEKCPDWQFPPSRSSVSPGRDKRRIKRSKRTENSLRRLRLPISNANL

>Lg c-type 7 c64057.graph_c0

MRISLVLPICLIVLFCGSARAKRFDTRCKLVRELGRVGIPNDHLLGQWVCLIEKVSNRDTRAFVVTPSGKKYYGLYQIPSRWCRSGKKGGECNIACESLLDDDIADDTACAVDIFHKEGFKYWNQWTVRCKNDDTITKEIYKCPDLNSPRSSPERELYADRLRKRRRLVRSKAQYIRQVYGVHA
